# Unveiling quasi-dark surface plasmon modes in Au nanoring cavities by cathodoluminescence

**DOI:** 10.1038/s41598-017-01607-5

**Published:** 2017-05-03

**Authors:** Chenglin Du, Wei Cai, Wei Wu, Yinxiao Xiang, Lei Wang, Mengxin Ren, Xinzheng Zhang, Jingjun Xu

**Affiliations:** The Key Laboratory of Weak-Light Nonlinear Photonics, Ministry of Education, School of Physics and TEDA Applied Physics Institute, Nankai University, Tianjin, 300457 China

## Abstract

Spectral resolving and imaging surface plasmon modes in noble metal nanostructures are important for applications in nanophotonics. Here, we use cathodoluminescence (CL) spectroscopy to excite and probe quasi-dark plasmon modes of Au nanoring cavities. Numerical simulations of both the spectra and the electromagnetic field distribution are carried out by using boundary element method. Good agreement between the experimental and simulated results is obtained. Particularly, CL is shown as an efficient method to probe quadrupole modes, which is difficult for traditional optical means. Moreover, a high Purcell factor in excess of 100 is obtained for the dark quadrupole modes in gold ring cavities. Our work provides an efficient way to explore the initial nature of surface plasmon modes in metal nanostructures.

## Introduction

Light-matter interaction at nanoscale has attracted much attention during the past decades because of its scientific relevance and potential applications^1^. Particularly, surface plasmons (SPs), the collective oscillations of electrons at the surface between a metal and a dielectric, can localize electromagnetic energy below diffraction limit^2, 3^. Besides, the plasmon resonance of metallic nanoparticles can be efficiently tailored by modifying their geometry due to the evanescent fields of SPs^4^. As a result, surface plasmon modes are vital ingredients for many applications such as nano-scale light sources^5^, photovoltaics^6^, and optical sensors^7, 8^. Therefore, it is critical for us to unveil and understand the plasmon modes of metallic nanoparticles experimentally. The spectrum properties of nanoparticles have been investigated extensively by far-field scattered light detection^9^, such as optical dark-field microscopy^10^, which provides excellent spectral resolution for nanoparticles. However, usually only radiative modes can be detected owing to the selection-rules imposed by the optical probe, as well as spatial resolution is limited by the diffraction limit. As an alternative, electron beams allow excitation of plasmons with fine spatial precision and retrieval of information on their local properties, with much higher spatial resolution down to few nanometer level^11, 12^. In this context, cathodoluminescence (CL) spectroscopy in which the emitted photons are detected^13–17^ and electron energy loss spectroscopy (EELS) through the detection of energy loss suffered by the inelastically scattered transmitted electrons^18–21^ are used to probe plasmons modes in metallic nanoparticles.

One of major differences between electron microscopy spectrum and conventional far-field optical spectrum is that dark plasmon modes can be detected by electrons^22, 23^, where dark modes corresponding to plasmon modes with low or zero dipole momentum^24^. A precise understanding of the difference between bright and dark modes is very important for plasmonic applications. Dark modes include quadrupolar and higher multipolar modes in single nanoparticles, coupled modes with vanishing dipole moments in nanoparticle pairs^25^, and propagating modes in nanoparticle chains^26^. Scattering is the dominant process for the bright modes, however, the dark modes are dominated by absorption which results in longer lifetimes and narrower spectral line widths^27^. The dark plasmons are indispensable elements for realizing Fano resonance^28^, and also can be ideal candidates for enhanced biological sensors and subwavelength high-Q optical cavities. Moreover, dark plasmons can couple with a localized dipole as strong as bright modes^29^, which can be applied to the emission control at nanoscale and the plasmonic nanolaser. Theoretically, absolute dark plasmons with zero dipole momentum can only be detected by EELS rather than CL, because only far-field emitted light can be collected in CL. However, due to the imperfection of samples or retardation effect, quasi-dark modes with low dipole momentum might be detected in experiments^30^. Until now dark plasmons have been studied a lot using EELS in coupled nanorods^22^, nanodisks^23^ and nanoparticles^31^. In contrast, It is always lack of direct experimental evidence whether CL can be used to probe dark plasmons.

In this paper, a gold nanoring cavity is chosen for CL experiments. Despite its geometric simplicity, the system has shown tunable plasmon properties and is desirable for a variety of applications^32, 33^. Moreover, to the best of our knowledge, the plasmon properties of this structure have not been explored using electron beams. Through numerical simulations by rigorous solving Maxwell equations using boundary element method (BEM)^34, 35^, the excited localized surface plasmon modes in Au nanoring cavities by an electron beam and a plane wave are compared. Then we use CL spectroscopy for direct observation both spectral and spatial properties of localized surface plasmon modes in the single Au nanoring cavity. On the basis of experimental and simulated data, we demonstrate that spatially resolved CL experiments allow us to directly probe dark surface plasmon mode on an Au nanoring cavity, which is hardly be realized by traditional optical excitation method. We also obtain the Purcell factor of *F*_*P*_ = 133 under experimentally measured quality factor *Q* = 4.7 for a *r* = 60 nm ring cavity. Our findings demonstrate not only the superiority of CL spectroscopy to investigate dark modes at nanoscale, but also potential applications of dark modes of Au nanoring cavities in spontaneous emission and optical antennas techniques.

## Results

**Figure 1:**
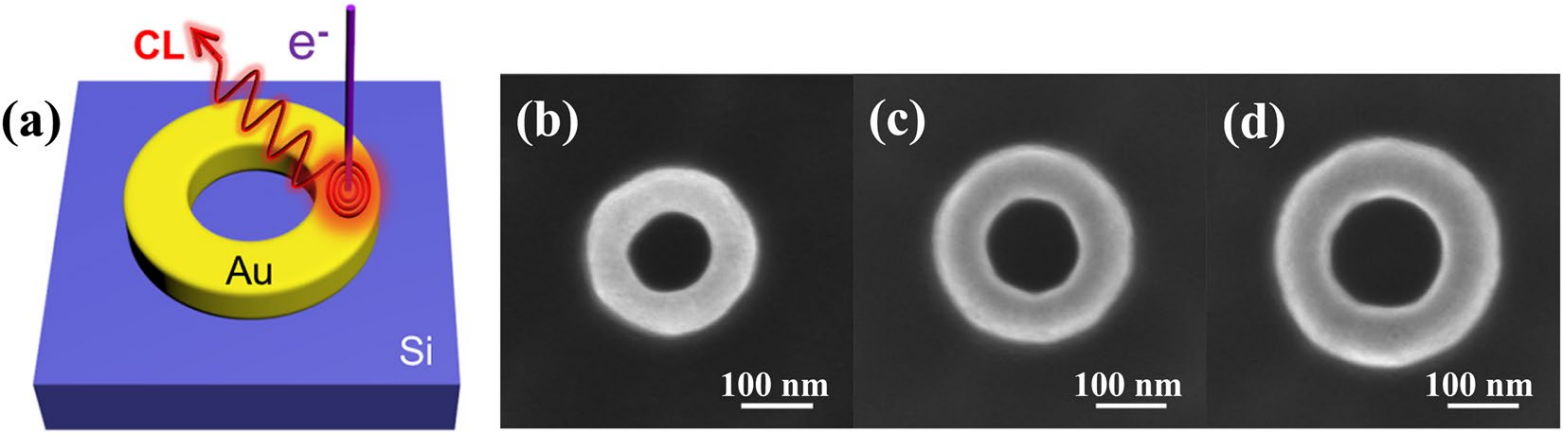
Schematic illustration and SEM images of the Au nanoring cavities. (**a**) Scheme of an Au nanoring cavity on a crystalline silicon substrate. The cavity is excited using electrons and then CL spectra are measured. (**b**–**d**) SEM images of our fabricated Au nanorings with different inside radii *r* = 60, 70 and 80 nm, and outside radii *R* = 2*r*.

**Figure 2:**
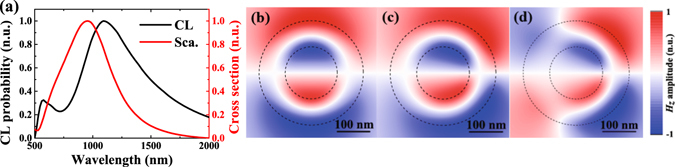
Comparison of the calculated CL and scattering spectra, and the normalized amplitude distributions. (**a**) Calculated CL emission spectrum (black) for a single Au nanoring with inside radius 80 nm and thickness 100 nm surrounded by vacuum, compared to the calculated scattering cross section (red) by plane wave excitation under normal incidence. (**b**) Calculated *H*_z_ field in *x*-*y* plane for *r* = 80 nm nanoring at *λ* = 954 nm (*m* = 1) by a plane wave excitation. (**c**) and (**d**) show the calculated *H*_z_ field in *x*-*y* plane for the same structure at *λ* = 1212 nm (*m* = 1) and 592 nm (*m* = 2) by 30 keV electron beam excitation, respectively. The electron beam was positioned at *r* = 120 nm and the cuts are taken at 10 nm above the upper surface. The black dashed circles indicate the edge of the structure.

A schematic drawing of the studied structure is presented in Fig. 1(a). Figure 1(b–d) show scanning electron microscope (SEM) images of the nanorings with different sizes. To understand the plasmon response of the fabricated nanoring cavities, the CL emission probability for the Au nanoring cavity of *r* = 80 nm excited by 30 keV electron beam at the position of *r* = 120 nm is simulated. For comparison, the scattering cross section for the same geometry excited by a plane wave under normal incidence is also shown in Fig. 2(a). As seen, for plane wave excitation, only one peak is observed centered at *λ* = 954 nm in 500–2000 nm wavelength range, while for electron excitation, apart from a strong resonant peak at *λ* = 1212 nm, another narrow peak appears at *λ* = 592 nm. To further identify the nature of the resonant peaks, the induced near-field distributions are calculated. Three panels in Fig. 2(b–d) show the *H*_*z*_ fields in the nanoring for each peak wavelength at 954, 1212 and 592 nm, respectively. One can find that a symmetric dipole mode (*m* = 1) is excited by a plane wave. In contrast, not only a slightly asymmetric dipole (*m* = 1) but also an obviously asymmetric quadrupole (*m* = 2) modes are excited by electron bombardment. For the same dipole mode, the resonant wavelength in scattering spectrum has a blue shift compared with the CL spectrum. The scattering spectrum is obtained from the plane wave far-field excitaiton, while CL is a kind of near-field excitation method. This discrepancy comes from the physics of a driven and damped harmonic oscillator^36, 37^. It is interesting to note that the CL technique enables probing of the quadrupole resonance. This is not possible with optical excitation under normal incidence and only the bright modes can be excited due to the selection rules. By contrast, due to the localized excitation nature of the electron beam, it has the ability to excite both bright and dark modes. Generally, the electron beam spot size is on the order of 1 nm and the radial extent of the evanescent electric field about the electron beam is on the order of 10 nm^16^. Therefore under electron beam excitation configuration, the retardation effect comes into play. Moreover, It is worth stressing that there are some energies leaking out from the nanoring cavity due to asymmetric excitation and retardation effect, as a result, this quadrupole mode can be detected by CL and can be named as a quasi-dark mode.

**Figure 3:**
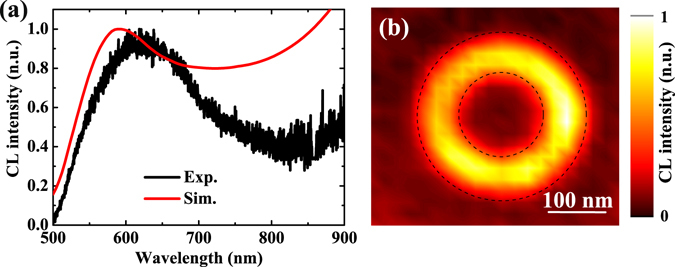
Comparison of the measured and calculated CL spectra, and the normalized CL map. (**a**) Measured (black) and calculated (red) CL spectra for *r* = 80 nm nanoring excited by 15 keV electrons at the position of *r* = 120 nm. (**b**) The CL intensity as a function of excitation position for the same nanoring at *λ* = 604 nm, integrated over a 20 nm bandwidth. The black dashed circle indicates the edge of the nanoring.

To verify our theoretical predictions, the experimental CL spectrum for a single Au nanoring cavity of *r* = 80 nm is plotted by the black curve in Fig. 3(a). Within the instrument detection limit of 500–900 nm spectra range, only quadrupole mode at 604 nm is detected. Compared to the simulated CL emission spectrum (red curve in Fig. 3(a)), the agreement between experimental and simulated results is quite satisfactory except a little redshift. This redshift comes from that the substrate effect is ignored in our simulations. Besides, It is also significant to study the spatial excitation profile of the nanoring. Figure 3(b) shows the spatial distribution at *λ* = 604 nm and a bright ring around the nanoring structure is observed. The quadrupole mode observed here is infinitely degenerate due to the rotational symmetry of the nanoring structure, which is why a bright ring appears in the excitation map when the electron beam is scanned over the sample.

**Figure 4:**
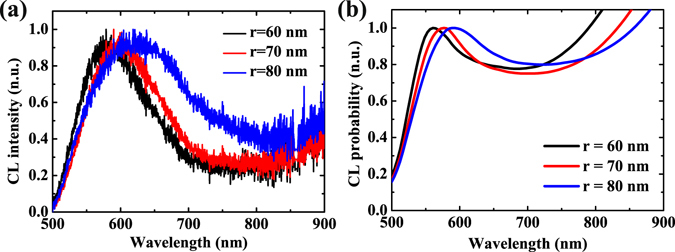
Comparison of the measured and calculated CL spectra with different radii. (**a**) Measured CL spectra for Au nanorings with different radii *r* = 60, 70 and 80 nm, the quadrupolar resonance wavelength *λ* = 563, 590 and 604 nm, respectively. (**b**) Calculated CL spectra for the geometries corresponding to the measurements of (**a**), the quadrupolar resonance wavelength *λ* = 561, 578 and 592 nm, respectively.

Figure 4(a) shows the measured CL spectra for nanoring cavities with different radii and a constant height of 100 nm. The spectra were obtained when the electron beam was incident at the position of (*r* + *R*)/2. The resonance wavelengths of quadrupole modes shift to longer wavelengths with increasing cavity radius from 60 to 80 nm, which depends strongly on the cavity size. In according to the CL experiments, the far-field emission spectra resulting from incoming electrons impinging on the ring structures were calculated by BEM and are shown in Fig. 4(b). The features seen in the calculated spectra are in good agreement with those of the experimental spectra, reproducing the shifts to longer wavelengths with increasing radius. Physically, this red shift can be accounted for as follows. With the increase in the cavity size, the separation between the dipole or quadrupole charges increases which reduces the restoring force and, consequently, the oscillation frequency decreases resulting in a red-shifted peak^38^. We note that for the same size of the Au nanoring, the experimental resonance wavelength has a slight red shift compared to the calculated one. We attribute this to the influence of substrate which increases the effective refractive index of environment.

**Figure 5:**
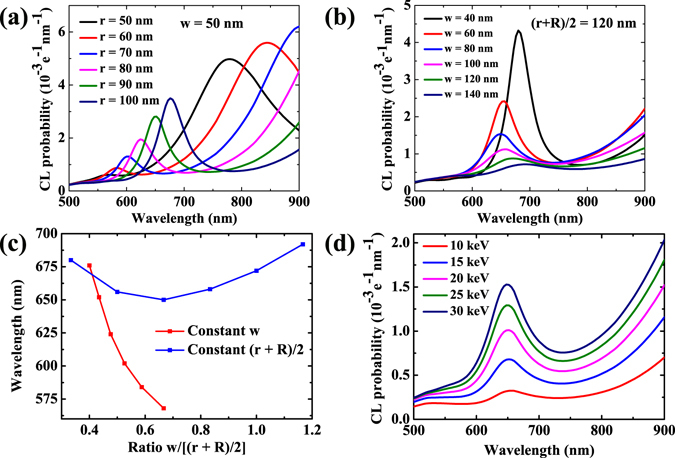
The influence of the ratio of the Au nanorings and the energy of electron beam on CL. The thickness of the Au nanoring *h* = 50 nm, the electron beam is incident at the position of (*r* + *R*)/2 and the energy of electron beam is 30 keV for (**a**–**c**). (**a**) Calculated CL spectra for nanorings with a constant width of 50 nm and different inside radii *r*. (**b**) Calculated CL spectra for nanorings with a constant effective radius of (*r* + *R*)/2 = 120 nm and different width *w*. (**c**) The relationship between the ratio parameter *w*/[(*r* + *R*)/2] and the resonant wavelength, which is extracted from (**a**) and (**b**). (**d**) The influence of the energy of electron beam for an *r* = 80 nm, *w* = 80 nm nanoring cavity.

The quadrupole mode in a nanoring cavity can be tuned flexibly because there are four parameters (inside radius *r*, outside radius *R*, width *w* and thickness *h*) in this kind of structure. Here we define a ratio parameter *w*/[(*r* + *R*)/2] and study the relationship between that and the resonant wavelength. The thickness *h* is set as 50 nm and the electron beam is incident at the position of (*r* + *R*)/2 throughout the following simulations. Figure 5(a) shows the calculated CL spectra for nanoring cavities with different inside radii and a constant width of 50 nm. The resonant wavelengths of quadrupole modes have red shifts and enhancements in CL probability with the radii increasing. For small-radius cavities, the dipole modes can also appear at long wavelength ranges. Then, the position of electron beam is fixed while the width is varying. In this situation the CL spectra seem different and the results are shown in Fig. 5(b). Though the width varies from 40 to 140 nm, the resonant wavelengths of quadrupole modes change a little. Obviously, the CL probabilities decrease with the width increasing which is attributed to the increase of absorption loss in metal structure. Figure 5(c) shows intuitively the relationship between the ratio parameter and the resonant wavelength under above-mentioned situations. It is noteworthy that the resonant wavelength is more sensitive to the effective radius (*r* + *R*)/2 than the width *w*. At last, the influence of the energy of electron beam is investigated and the result is shown in Fig. 5(d). For the same structure, when the energy increases from 10 to 30 keV, the resonant wavelength of the quadrupole mode does not shift but the CL probabilities increase gradually.

Finally, it is significant to estimate the Purcell factor for the quadrupole modes in the nanoring cavities, because current works show that dark modes are of essential importance for the spectroscopic application with high local field. The Purcell factor of the cavity can be calculated by ${F}_{P}=\frac{3}{4{\pi }^{2}}\frac{Q}{V}{(\frac{\lambda }{n})}^{3}$, where the quality factor *Q* can be obtained by experimental obtained spectra, *n* is the reflective index of the cavity medium and the mode volume *V* can be calculated numerically. For the *r* = 60 nm Au nanoring cavity, from the near-field, we have calculated the mode volume^39^, which is found to be 0.00269 ${\lambda }_{0}^{3}$. With a known calculated quality factor of *Q* = 7.2, we find a Purcell enhancement factor of *F*_*P*_ = 204. Using the quality factor *Q* = 4.7 obtained in experiments, a Purcell factor of *F*_*P*_ = 133 is expected.

## Discussion

In this report, we have applied CL spectroscopy combined with an SEM to investigate the plasmonic behavior of Au nanoring cavities for the first time, and a quasi-dark quadrupole mode depending on the size is observed. In addition, simulated CL spectra using the retarded BEM are agreement with the experimental results. It is also worth noting these dark modes can not be accessed by traditional optical excitation under normal incidence. Furthermore, on the basis of experimental and simulative data, we demonstrate that spatially resolved CL experiments allow us to directly image dark surface plasmon mode on an Au nanoring cavity with resolution less than 40 nm. The influences of the ratio of the Au nanorings and the energy of electron beam on CL have been investigated as well. We find that the resonant wavelength is more sensitive to the effective radius (*r* + *R*)/2 than the width *w* and only the amplitude of the CL is affected by the energy of the electron beams. Finally, a quality factor of *Q* = 4.7 is found for the 60 nm inside radius cavity, hence a resulting Purcell factor around 133 is obtained. We expect that these conclusions can be applied in control of spontaneous emission and light-matter interaction in general, paving the way for nanoplasmonic devices.

## Methods

Au nanoring cavities were prepared as follows. Firstly an Au film about 100 nm thick was sputtered onto a crystalline silicon substrate using ion beam. Then we used the 30 keV Ga^+^ beam of a focused ion beam (FIB) system (Helios NanoLab 600i, FEI) to carve ring structures with inside radii *r* = 60, 70 and 80 nm, and outside radii *R* equates to 2*r*, respectively.

We used CL spectroscopy to excite plasmon modes in the Au nanoring cavity and measured the emission in the far field. The samples were excited by the 15 keV electron beam of an SEM, which was focused to a 6 nm spot onto the sample surface. A parabolic mirror placed above the sample collects the light emitted from the sample and guides it to a spectrometer in which the light is spectrally resolved and detected using a liquid-nitrogen-cooled charge-coupled device array, recording spectra in the 500–900 nm wavelength range. The measured spectra were corrected for system response by measuring the transition radiation spectrum for gold and comparing that to theory^40^.

Additionally, we performed simulations by solving Maxwell equations for this system using the BEM for rotationally invariant structures. The fields in the BEM calculations can be separated into azimuthal components with a dependence on the azimuthal angle *ϕ* as e^*imϕ*^, where *m* *=* 0, ±1, ±2, … is the azimuthal number. In the calculation, contributions up to an order |*m*| ≤ 5 were taken into account, high enough for the results to reach convergence. The calculations are performed using tabulated optical constants for Au^41^. The ring cavity has three parameters: inside radii *r* = 60, 70 and 80 nm, outside radii *R* = 2*r* and thickness of 100 nm which are the same as experimental ones. The curvature of the corners is set as 10 nm.
